# Acute Abdominal Pain With Gastrointestinal Wall Thickening and Edema: Insights From Two Case Reports

**DOI:** 10.7759/cureus.79087

**Published:** 2025-02-16

**Authors:** Jiaming Lei, Ling Wu

**Affiliations:** 1 Department of Gastroenterology, People’s Hospital of Leshan, Leshan, CHN; 2 Department of Cardiology, The Affiliated Hospital of Southwest Medical University, Luzhou, CHN

**Keywords:** abdominal pain, acute gastroenteritis, fever, gastrointestinal edema, infection

## Abstract

Gastrointestinal diseases encompass a wide range of etiologies, including tumor-related, infectious, ischemic, immune-mediated, and vascular conditions, all of which can present with similar imaging features. Among these, phlegmonous gastroenteritis is a rare, acute gastrointestinal infection characterized by purulent inflammation in the submucosal and muscular layers of the gastrointestinal wall. Clinical manifestations typically include sudden abdominal pain, nausea, vomiting, fever, and abdominal distension, with imaging showing gastrointestinal wall thickening and stromal edema. The disease’s nonspecific clinical and imaging features often lead to misdiagnosis. This report describes two cases of acute abdominal pain with gastrointestinal wall thickening and edema, providing insights into the early identification of phlegmonous gastroenteritis. Both patients recovered completely after empirical antibiotic therapy, highlighting the importance of early imaging diagnosis, endoscopy, and histopathological analysis in managing this rare condition. Early recognition and intervention are essential to prevent severe complications and improve patient outcomes.

## Introduction

Gastrointestinal diseases have a wide range of causes and can be broadly classified based on their underlying mechanisms, including tumor-related, infectious, ischemic, immune-mediated, vasculitis, vascular edema, and pressure-related etiologies. Examples include inflammatory bowel disease, autoimmune enteritis, ischemic colitis, and eosinophilic gastroenteritis [1-5]. Rare diseases, such as hereditary and acquired angioedema, should also be considered in differential diagnoses, especially when episodic severe abdominal pain and transient intestinal edema are observed [6]. With the increasing use of imaging techniques, particularly computed tomography (CT), the clinical detection of gastrointestinal wall edema has become more common. These diseases often share similar imaging features, such as gastrointestinal edema, lumen narrowing, and inflammation of surrounding tissues, which can complicate diagnosis and management, making them both complex and challenging.

Phlegmonous gastroenteritis, a relatively rare acute gastrointestinal infection, typically presents with sudden abdominal pain, nausea, vomiting, bloating, and fever. Imaging may show thickening of the gastrointestinal wall and stromal edema [7]. The exact etiology of the disease remains unclear, and its clinical and imaging presentations are highly nonspecific, often confusing it with other gastrointestinal diseases. However, it progresses rapidly and has a high mortality rate, making early imaging diagnosis, endoscopy, and histopathological analysis particularly important for understanding and managing the disease.

This report presents two cases of acute abdominal pain associated with gastrointestinal wall thickening and edema, aimed at exploring the clinical features, imaging findings, and diagnostic strategies for early phlegmonous gastroenteritis. We emphasize the importance of a systematic diagnostic approach in accurately identifying such rare diseases and call for further efforts to enhance the early recognition and timely treatment of gastrointestinal inflammatory diseases to improve patient outcomes.

## Case presentation

Case 1

**Figure 1:**
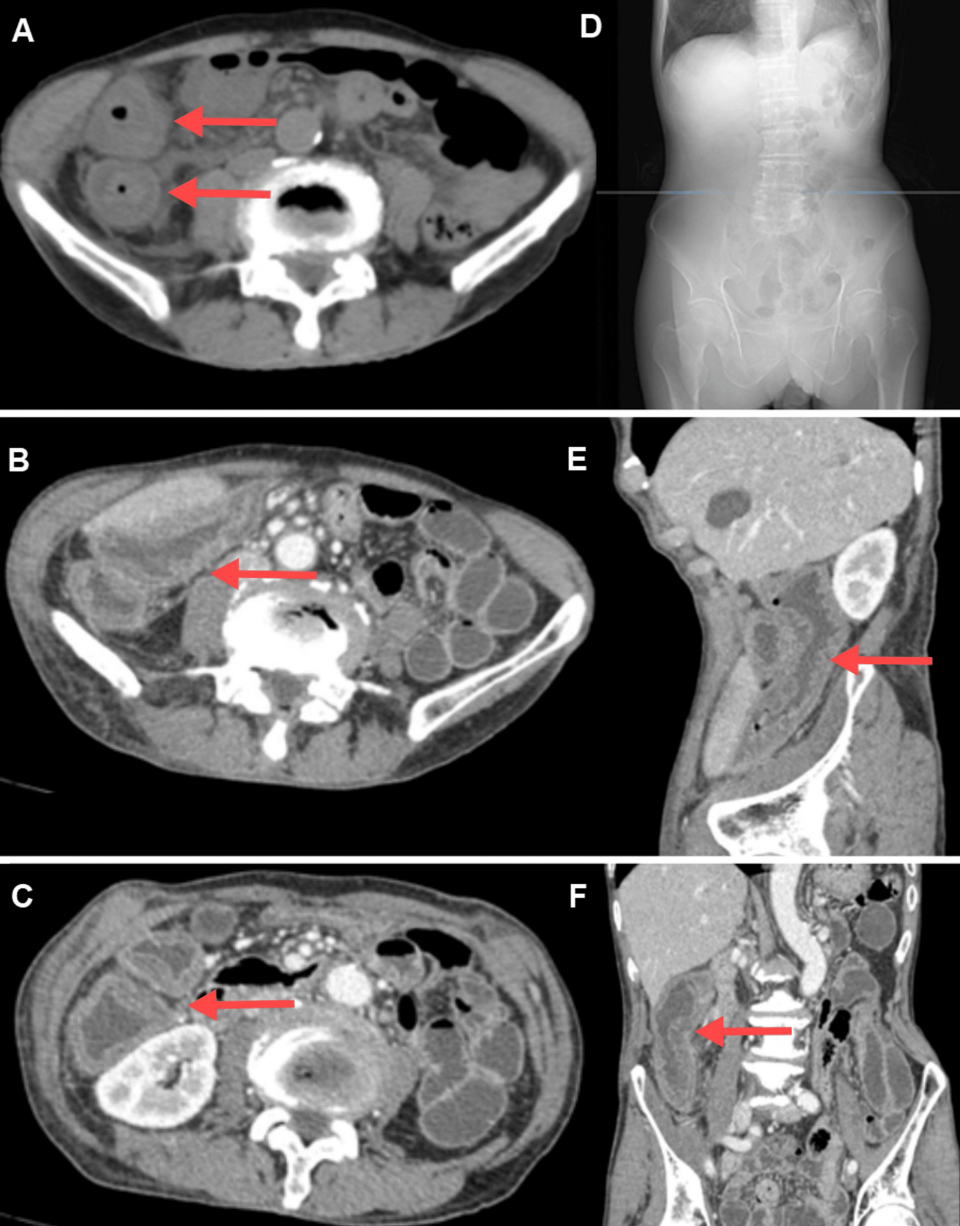
CT Imaging of Colonic Wall Thickening and Edema in the Ascending Colon and Ileocecal Region (Red arrow point). (A) Non-contrast CT shows significant wall thickening in the ascending colon in the right mid-abdomen, with luminal narrowing and a maximum wall thickness of approximately 13 mm. A faint “double halo” sign is visible (left). (B) Contrast-enhanced CT at the hepatic flexure of the colon (left). (C) Contrast-enhanced CT at the ascending colon (left). (D) The blue line indicates the level of panel A on the X-ray image, which shows no air-fluid levels or signs of obstruction. (E) Sagittal view.

A 70-year-old male presented with a three-day history of sudden, persistent cramping pain in the right lower abdomen, accompanied by nausea, abdominal distension, and fever, but without diarrhea or mucus/bloody stool. The patient had no significant past medical history or family history of gastrointestinal diseases. Abdominal examination revealed marked tenderness in the right lower quadrant. Non-contrast CT revealed significant edema and thickening of the intestinal wall in the right transverse colon, descending colon, and ileocecal region, with a maximum wall thickness of 13 mm. Additional findings included luminal narrowing, rough serosal surfaces, and scattered exudates and fluid in the peritoneal cavity and adjacent retroperitoneal space (Figures 1A-1D).

Laboratory findings

**Figure 2:**
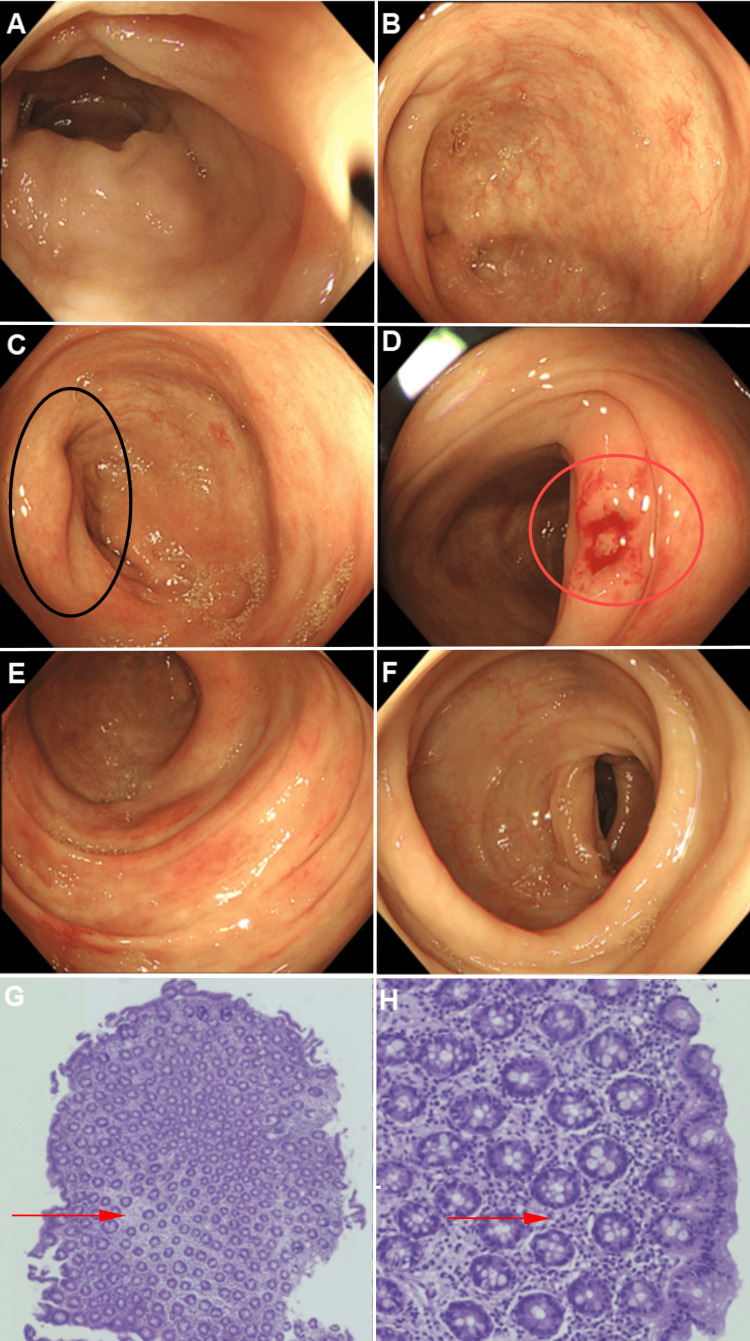
Endoscopic Images of Colonic Mucosa and Histopathological Findings from Biopsy Samples. (A) Terminal ileum: Mucosa appears smooth and swollen without hyperemia or erosions. (B) Cecum: No evidence of ulceration, mass lesions, or erosion. (C) Ileocecal valve: Mucosal swelling is noted (Black ellipse shown). (D) Ascending colon biopsy site: Thickened mucosal layers without abnormalities (Red ellipse shown). (E) Transverse colon: Smooth mucosa with mild edema. (F) Descending colon: No pathological findings apart from mucosal swelling. (G) Low-power view (H&E stain, ×5) of ascending colon biopsy reveals chronic mucosal inflammation with prominent stromal edema and no tumor cells. (H) High-power view (H&E stain, ×20) confirms the findings, with no significant lymphocytic, neutrophilic, or eosinophilic infiltration (Red arrow shown).

Total white blood cell count of 4.97 × 10^9^/L, hemoglobin concentration of 151 g/L, and neutrophil percentage of 86.5%. High-sensitivity C-reactive protein (hsCRP) was elevated at 155.51 mg/L, and procalcitonin (PCT) was significantly increased at 13.110 ng/mL. Tests for IgG4, C4, carcinoembryonic antigen (CEA), blood culture, and stool culture were unremarkable. Contrast-enhanced CT demonstrated symmetric thickening and edema of the right transverse colon, ascending colon, and ileocecal region, with surrounding peritoneal and retroperitoneal exudation and fluid accumulation. No abnormal enhancement masses, mesenteric vascular abnormalities, or lymphadenopathy were observed (Figures 1B-1F). Endoscopy: Colonoscopy showed significant swelling of the ileocecal valve and ascending colon with smooth, edematous mucosa. The colonic folds appeared flattened, with no erosions, ulcers, or masses but localized vascular dilation. Biopsy revealed thickened mucosal layers and prominent submucosal edema (Figures 2A-2F).

Histopathology

Demonstrated chronic mucosal inflammation and stromal edema, with no evidence of tumor cells or significant lymphocytic or neutrophilic infiltration (Figures 2G-2H). The patient was treated with anti-infective therapy and fluid replacement. Follow-up tests showed significant improvement in hsCRP (66.37 mg/L) and PCT (0.630 ng/mL). During a two-month follow-up, the patient reported no further episodes of abdominal pain, and repeat CT imaging showed complete resolution of gastrointestinal wall thickening and edema.

Case 2 

**Figure 3:**
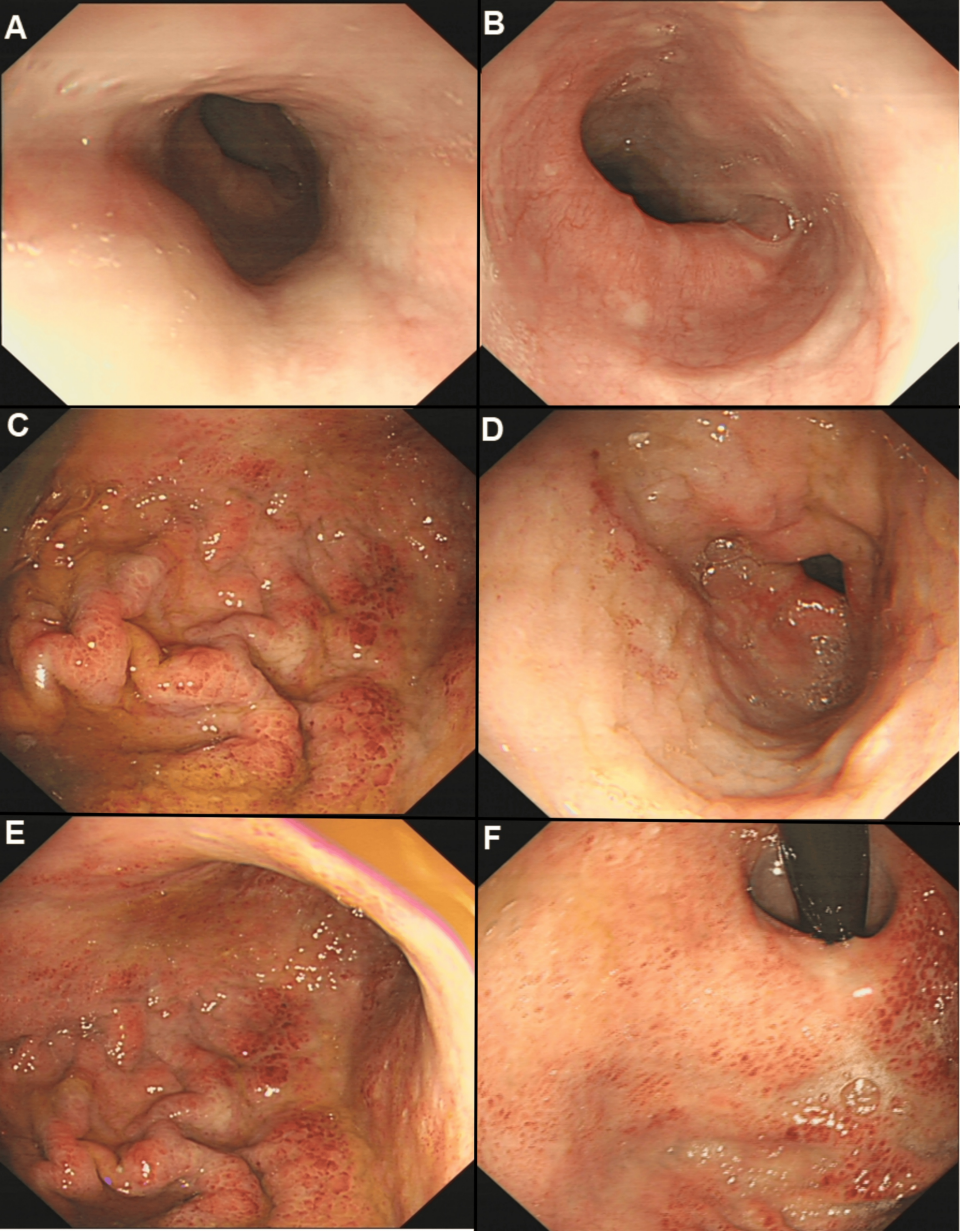
CT Imaging of Esophageal and Gastric Wall Thickening and Edema and Histopathological Findings from Biopsy Samples. (A) Mid-esophagus: Smooth mucosa with a tense, swollen appearance and no signs of erosion or ulceration. (B) Distal esophagus: Similar smooth mucosal surface without masses. (C) Greater curvature of the upper gastric body: Mild focal redness and swelling of mucosa. (D) Antrum: Flattened folds with mucosal edema. (E) Greater curvature of the mid-gastric body: Swelling without pathological erosion or ulceration. (F) Cardia: Edematous mucosa with no evidence of malignancy or inflammatory ulcerations.

A 70-year-old male presented with a two-day history of persistent upper abdominal pain, intermittently exacerbated, accompanied by nausea, vomiting, chills, and fever, with a maximum temperature of 39.2°C. He denied any history of consuming contaminated food, medications, toxic substances, or foreign body ingestion. Non-contrast CT revealed uneven thickening and edema of the distal esophagus and gastric body, along with peritoneal effusion and mesenteric edema. Endoscopy showed that the esophageal mucosa was smooth and normal in color, with a clear vascular network. However, the mucosa appeared notably tense and swollen when submerged in water, with no stenosis or rigidity in the lumen. The gastric mucosa exhibited significant edema and thickened folds, with multiple focal areas of redness but no evidence of erosion, ulceration, or masses. The urea breath test for Helicobacter pylori was negative (Figures 3A-3F).

**Figure 4:**
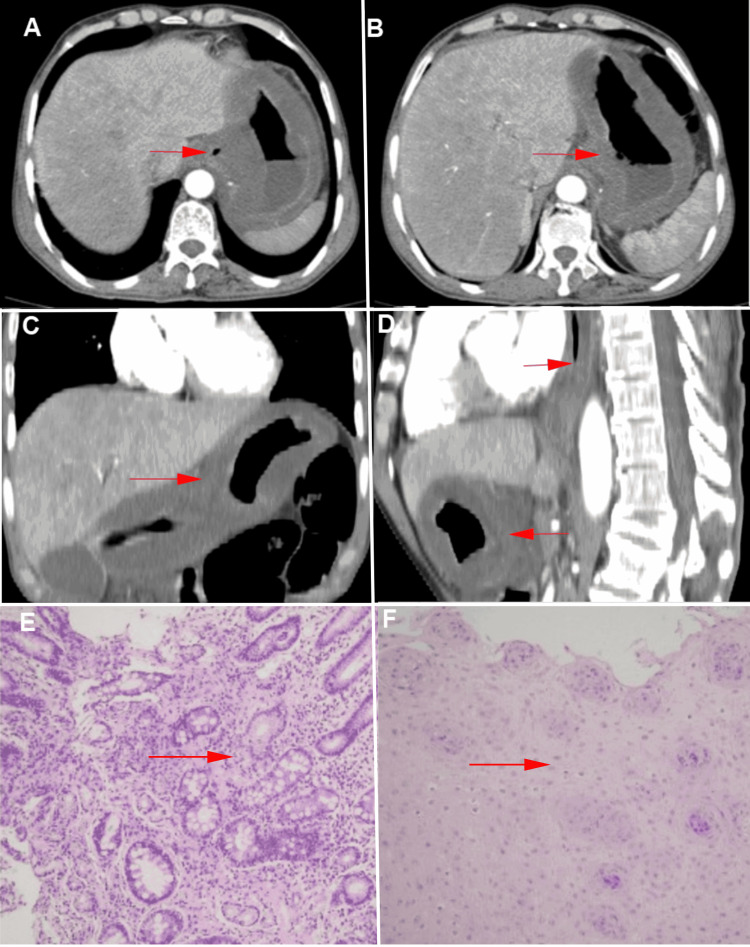
CT Imaging of Esophageal and Gastric Wall Thickening and Edema and Histopathological Findings from Biopsy Samples. (A) Contrast-enhanced CT of the upper gastric body shows thickened walls without mass-like enhancement (red arrow). (B) The lower gastric body shows similar findings (red arrow). (C) Coronal view demonstrates diffuse gastric wall thickening (red arrow). (D) The sagittal view highlights wall edema without significant abnormal masses (red arrow). (E) Low-power view (H&E stain, ×10) of gastric body biopsy demonstrates chronic inflammation with stromal edema, without tumor cells or significant inflammatory cell infiltration. (F) Low-power view (H&E stain, ×10) of esophageal biopsy shows squamous epithelial hyperplasia and thickening, with no atypical cells or malignancy observed (red arrow).

Laboratory tests showed a total white blood cell count of 1.49 × 10^9^/L, an absolute neutrophil count of 1.23 × 10^9^/L, and elevated levels of hsCRP (331.75 mg/L) and PCT (67.800 ng/mL). Liver and renal function tests, urinalysis, stool occult blood testing, infection markers, serum lipids, glucose, cardiac enzymes, amylase, and tumor markers were within normal limits. Blood and stool cultures and stool bacterial and fungal smears were negative. Contrast-enhanced CT indicated diffuse thickening and edema of the middle to lower esophageal segments and the entire stomach. The gastric and esophageal walls showed layered enhancement without abnormal enhancement or mass lesions. Additional findings included scattered peritoneal effusion, mesenteric edema, and omental thickening (Figures 4A-4D).

Histopathological examination of a gastric biopsy revealed chronic mucosal inflammation and stromal edema without malignancy or significant infiltration by lymphocytes, neutrophils, or eosinophils. An esophageal biopsy showed squamous epithelial hyperplasia and thickening without atypical cells (Figures 4E-4F). The patient was treated with anti-infective therapy, acid suppression, supportive care, and nutritional supplementation. Symptoms improved, and he was discharged after achieving full recovery of oral intake. During a two-month follow-up, the patient remained asymptomatic, and repeat CT imaging confirmed the resolution of esophageal and gastric wall edema.

## Discussion

This report describes two cases of acute abdominal pain involving both the upper and lower gastrointestinal tracts, with both presenting severe abdominal pain and fever. Imaging showed extensive, symmetrical, and significant gastrointestinal wall edema and thickening alongside elevated infection markers. Pathological examination revealed stromal edema without evidence of malignancy or immune involvement. These cases provide unique insights into the early identification of phlegmonous gastroenteritis's clinical features despite the fact that gastrointestinal wall thickening is not uncommon in CT imaging. However, the characteristics of the two cases presented in this report - acute onset, bowel wall edema and thickening (in the submucosal layer), with the mucosal layer remaining intact, and severe systemic signs of infection - clearly differentiate them from common infectious gastrointestinal diseases.

In both patients, the mucosal layer of the gastrointestinal tract remained intact, and stool pathogen tests did not reveal any bacterial infection. This suggests that the etiology of these patients was not typical bacterial gastroenteritis but rather aligned with the features of phlegmonous gastroenteritis. Both patients fully recovered following empirical antibiotic treatment, further supporting the possibility of phlegmonous gastroenteritis in this clinical context.

Phlegmonous gastroenteritis is a rare condition characterized by purulent inflammation in the submucosal and muscular layers of the gastrointestinal wall, and clinical manifestations may include abdominal pain, fever, nausea, vomiting, bloating, and even severe complications such as septic shock and death. Although several hundred sporadic cases have been reported since Cruveilhier first described the disease in the 18th century, early diagnosis remains difficult, and there is a lack of clear diagnostic and management protocols [8].

These cases suggest that the primary infection source in phlegmonous gastroenteritis arises within the gastrointestinal tract, manifesting as thickening of the gastrointestinal wall, mucosal edema, and possible involvement of surrounding serosal layers. Early diagnosis and differential diagnosis often require a comprehensive evaluation of the patient’s history, physical examination, microbiological tests, laboratory markers, imaging findings, endoscopic examination, and histopathological analysis. Imaging plays a crucial role in the differential diagnosis of gastrointestinal wall thickening and edema. CT imaging typically shows symmetrical thickening of the gastrointestinal wall, layered enhancement, and changes in surrounding tissues, with the “double ring sign” or “target sign” being a typical imaging feature, reflecting contrast enhancement between the mucosa and muscle layers separated by the edematous submucosal layer [9-10]. This supports the pathophysiological characteristics of phlegmonous gastroenteritis. Endoscopy is useful in detecting mucosal changes such as erosion, ulcers, hyperplasia, vascular changes, and glandular abnormalities, which are crucial for further clarifying the diagnosis. However, in early phlegmonous gastroenteritis, endoscopic biopsy may fail to yield positive results. Some studies suggest that biopsy with a larger tissue sample using a snare technique may have a higher positive rate. Additionally, pathogen detection plays a key role, particularly next-generation sequencing (NGS), which has shown unexpected utility in disease diagnosis.

The pathogenesis of phlegmonous gastroenteritis is not yet fully understood, but it may be caused by local or disseminated infections, with pathogens such as gas-producing bacteria being potential culprits. CT imaging typically reveals thickening, low density, and layered enhancement of the gastrointestinal wall, and in some cases, gas-producing bacterial infections may result in intra-wall gas. The gut microbiome may also be involved in the pathogenesis, with complex interactions between factors such as altered intestinal permeability, activation of inflammatory cytokines, and changes in the gut microbiota [11]. The stromal edema observed in these cases may be caused by vascular leakage, immune activation, and dysbiosis. However, relying solely on CT imaging is often insufficient for a definitive diagnosis and must be combined with endoscopic pathological analysis. Histopathological examination shows significant edema, neutrophil, and plasma cell infiltration in the submucosal and muscular layers of the gastrointestinal wall, with even hemorrhage, necrosis, and thrombosis within the wall [12]. Despite this, phlegmonous gastroenteritis generally responds well to empirical antibiotic therapy, with patients recovering fully after treatment. Therefore, the treatment for phlegmonous gastroenteritis primarily relies on conservative antibiotic therapy. However, without early diagnosis and appropriate management, it can lead to severe complications, including peritonitis, mediastinitis, gastrointestinal perforation, sepsis, and even death [13]. Thus, early identification and timely intervention are crucial.

In summary, these cases highlight the challenges of diagnosing phlegmonous gastroenteritis as a rare but potentially fatal gastrointestinal disease. Although its pathogenesis is not fully understood, early recognition and treatment of this disease can significantly reduce the risk of disease progression. Furthermore, future research should focus on a deeper understanding of its pathogenic mechanisms to improve early diagnosis and treatment strategies, ultimately minimizing complications and mortality.

## Conclusions

Phlegmonous gastroenteritis, though rare, presents a significant diagnostic challenge due to its nonspecific clinical and imaging features, which overlap with other gastrointestinal diseases. The cases presented in this report demonstrate the importance of a comprehensive diagnostic approach, combining clinical evaluation, imaging, endoscopy, and histopathological analysis to accurately identify this condition. Despite its rare occurrence, early recognition and timely treatment with empirical antibiotics can lead to full recovery and prevent severe complications such as perforation, sepsis, and death. Further research into the disease's pathogenesis is necessary to improve diagnostic protocols and treatment strategies, ultimately reducing complications and mortality associated with phlegmonous gastroenteritis.
